# Ion transport in biological ion channels beyond classical electrostatics. Nanoscale confinement, non-linear concentration patterns and interfacial effects

**DOI:** 10.1007/s12551-026-01413-2

**Published:** 2026-02-10

**Authors:** María Queralt-Martín, Laidy M. Alvero-González, D. Aurora Perini, Elena García-Giménez, Antonio Alcaraz

**Affiliations:** 1 Laboratory of Molecular Biophysics, Department of Physics, University Jaume I, 12071 Castellón, Spain; 2 Instituto de Ciencia Molecular, Universidad de Valencia, Catedrático José Beltrán-2, 46980 Paterna, Spain

**Keywords:** Electrostatics, Ion transport, Nanochannel, Membrane proteins, Phospholipid membranes

## Abstract

Membrane transport has been the subject of intense investigation during decades in diverse scientific disciplines like physical chemistry, biophysics, and electrochemistry. While simplified models derived from continuous electrostatics generally hold, nanometer-sized channels often exhibit unexpected behaviors that stem from a variety of factors including the molecular nature of the solvent or the charge-charge correlations involving both the permeating ions and fixed charges within the membrane-pore system. In this short review, we summarize recent work focusing on two different salt concentration regimes with behaviors that contradict classical models. First, at low concentrations, critical interfacial effects such as access resistance occur involving a non-trivial interplay between membrane and protein charges. Second, in concentrated solutions, many ion transport properties show non-monotonic concentration patterns compatible with underscreening. Understanding these nanoscale deviations from typical microscale behavior is essential for accurately predicting the function of biological ion channels and designing advanced, atomic-sized fluidic devices for industry.

## Introduction

The description of ion transport through membrane nanochannels is particularly complex because of different distinctive factors. On the one side, the surface to volume ratio at the nanoscale is high enough so that the interactions between permeating ions and pore charges (the so-called *inner surface* effects) become dominant in contrast to what happens in microfluidics, where most properties are well described using *bulk* characteristics of fluids (Schoch et al. 2008; Bocquet and Charlaix 2010; Aluru et al. 2023). On the other side, transport is significantly impacted by interfacial effects at the pore/solution boundaries arising from the presence of membrane charges at the pore entrances (the so-called *outer surface* effects) and the geometrical constriction of current lines entering the pore (access resistance) (Hall 1975; Rostovtseva et al. 1998; Aguilella-Arzo et al. 2005; Alcaraz et al. 2017; Queralt-Martín et al. 2018). The rationalization of these traits in terms of available theoretical approaches becomes challenging when considering the effect of electrolyte concentration. Although most of the effects have been described in solutions mimicking physiologically relevant environments (extracellular fluid is ~ 0.15 M) (Liu et al. 2024; Smith, Lee, and Perkin 2016), even in these conditions, the strong nanoconfinement of biochannels yields relatively high local concentrations in the vicinity of protein and membrane charges to assure global electroneutrality (Helfferich 1962; Laksminarayanaiah and Lakshminarayanaiah 1969).

Hence, the constrained and often irregular geometry of biological nanochannels, which typically involve corrugated amphoteric surfaces, intrinsically contains a variety of electrolyte conditions. These different environments affect solvent properties (dielectric saturation of water) and solute density, including also significantly entropic forces pushing ions towards less-confined regions (Aguilella-Arzo et al. 2009; Malgaretti et al. 2013; Berezhkovskii et al. 2015; Queralt-Martín et al. 2018; Alvero-González et al. 2024).

As noted by abundant literature, concentrated solutions require more elaborated approaches than the classical Debye-Hückel theory, which relies on the linearization of the Poisson-Boltzmann equation and assumes that ions are point charges in a structureless uniform solvent (Bockris and Reddy 1998; Avni et al. 2022; Robertson et al. 2023; Elliott et al. 2024). Among others, ion-related factors (finite size, excluded volume, ion correlations) as well as solvent-associated effects (dielectric friction, fluid advection, and their coupling with ion electrodiffusion) should be taken into account (Dufrêche et al. 2005; Zhang et al. 2020; Avni et al. 2022; Robertson et al. 2023; Tang et al. 2024).

Here, we review recent investigations into ion permeation through diverse nanochannels and other membrane systems, revealing that ionic transport is regulated by salt concentration in such a way that two separate concentration regimes are visible. In dilute solutions (low concentration), ion transport is dominated by interfacial effects. This aligns with classical Debye screening models, once the complex interplay between the inner pore charges and outer membrane charges is considered (Alvero-González et al. 2024; Queralt-Martín et al. 2026). Conversely, in concentrated solutions (high concentration), the observed ion transport patterns deviate significantly from classical models, showing non-monotonic re-entrant behavior indicative of underscreening (Robertson et al. 2023; Queralt-Martín et al. 2023; Elliott et al. 2024).

A critical analysis using diverse electrochemical quantities (channel conductance, noise, activation energy, selectivity) shows that the transition between these two regimes is not universal. Instead, it is system- and quantity-dependent, meaning that the crossover point changes depending on the specific nanochannel and the property being measured. This variability occurs because several factors are at play, including ionic diffusion, ion-ion interactions (how ions interact with each other), and ion-pore interactions (how ions interact with the channel walls). These factors contribute differently to each measurement, which is why a single, simple model fails to capture the full behavior across all conditions.

The implications of the research gathered here transcend the biophysical characterization of ion channels, providing a fundamental basis for understanding the behavior of electrolyte solutions utilized in various membrane systems. This knowledge is critical across a spectrum of technologies (Liu et al. 2024; Emmerich et al. 2024; Chantipmanee and Xu 2024; Iarossi et al. 2025). Specifically, moderately concentrated electrolytes demonstrate considerable potential in domains such as electrochemical reactors, water desalination, and bioelectrochemistry. Conversely, a distinct class of extremely concentrated solutions is indispensable for specialized technological applications, including supercapacitors, rechargeable batteries, and high-density energy-storage devices (Pletcher 2008; Vidakovic-Koch et al. 2012; Perry et al. 2020; Gao et al. 2022; Sharkh et al. 2022; Queralt-Martín et al. 2023).

## Concentration-dependent regimes for ion transport in biological nanochannels

**Fig. 1:**
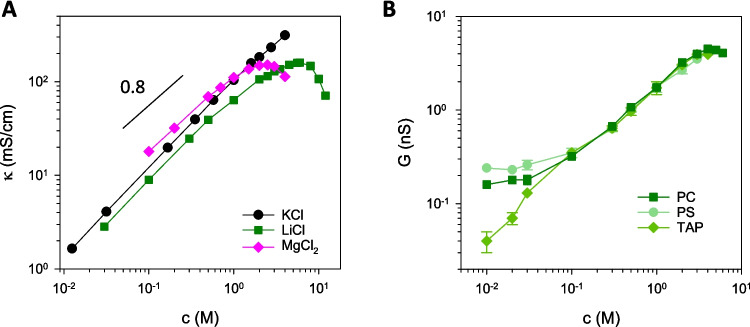
**A** Solution conductivity for KCl, LiCl, and MgCl_2_ as a function of electrolyte concentration. **B** Measured conductance scaling with LiCl salt concentration of the OmpF porin in three differently charged membranes (neutral PC, negative PS, and positive TAP)

Investigating how certain electrochemical properties (channel conductance, selectivity, noise) change as a function of electrolyte concentration is a common way to evaluate how mobile ions screen the fixed charges existing in the system (Alcaraz et al. 2004; Schoch and Renaud 2005; Schoch et al. 2008; López et al. 2009; Verdiá-Báguena et al. 2012; Queralt-Martín et al. 2015). In order to separate the activity of the channel itself from the electrolyte flowing through it, the study of electrolyte conductivity *κ* as a function of concentration seems a necessary initial step (Shcherbakov et al. 2021; Queralt-Martín et al. 2023). Figure 1A shows that some electrolytes like KCl display a quasi-linear *κ* vs *c* relationship up to their solubility limit, whereas others like LiCl and MgCl_2_ show clear non-linearities beyond molar concentration. DHO theory predicting *κ* ~ *c* fails in concentrated solutions due to a number of factors including long-range ion-ion correlations and solvent-excluded volume (Adar et al. 2018; Avni et al. 2022).

The question that follows naturally is whether solution conductivity, which is a *bulk* property of the solution, is a fair indicator of what to expect within a nanochannel, where fluid is confined within charged *surfaces*. On the one hand, surface charge effects should be visible in the low-concentration regime, where ion screening of charges (either inner surface or outer surface ones) is less efficient. On the other hand, it is not straightforward to anticipate if the transition between the low-concentration Debye screening regime and the underscreening high-concentration regime (Elliott et al. 2024) is universal or depends on the electrochemical property under study (conductance, selectivity, noise, etc.). In this sense, it is known that each measured property reflects a different balance between electrostatic exclusion, ionic diffusion, and potential binding effects (Alcaraz et al. 2009; Queralt-Martín et al. 2015), so it seems reasonable that something similar happens as regards nanoscale confinement, charge-charge correlations, and solvent properties.

Figure 1B shows the measured channel conductance of OmpF porin in three different membranes (neutral PC, negative PS, and positive TAP) as a function of LiCl concentration (measurements were performed as described elsewhere Queralt-Martín et al. 2023). Three different regions are visible in the same plot. In dilute solutions, interfacial effects dominate, since it is shown that membrane surface charges crucially determine the overall conductance (Alvero-González et al. 2024; Queralt-Martín et al. 2026). Around physiological conditions and up to moderately concentrated solutions, channel conductance scales roughly with electrolyte concentration, as predicted by classical models such as Debye screening and Donnan equilibrium. At high concentrations (see Fig. 1B), the linearity between *G* and *c* is lost and biphasic re-entrant concentration patterns appear (Queralt-Martín et al. 2023; Elliott et al. 2024). The following sections will explain in detail the origin of these separate regions by comparing different nanometric channels.

## Diluted solutions: scaling behavior in conductance-concentration relationships and permeability-selectivity trade-off

Among the transport properties in membrane pores, the power-law relationship between channel conductance—expressed as *G* ~ *c*^*α*^ (known as concentration scaling behavior)—has garnered particular attention because it allows a direct comparison with electrolyte conductivity (Secchi et al. 2016; Queralt-Martín et al. 2018; Noh and Aluru 2020).

**Fig. 2:**
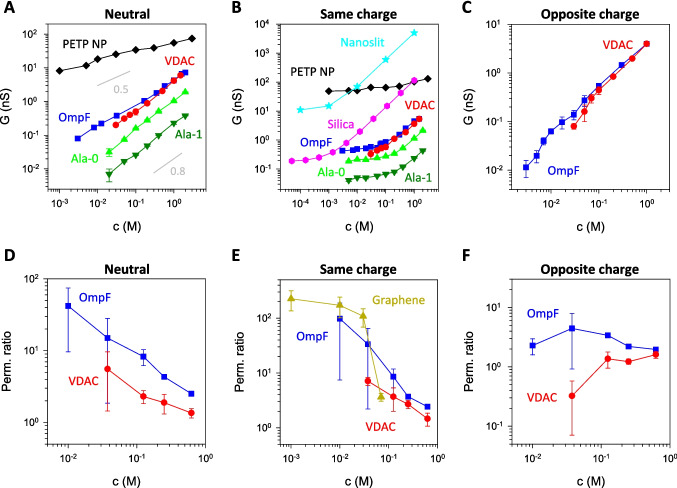
Tuning of concentration scaling by outer surface charges. **A**–**C** Measured pore conductance as a function of salt concentration for various biological ion channels and nanopores: polyethylene terephthalate nanopores (PETP NP) from Lev et al. (1993); nanoslits from Schoch and Renaud (2005); silica channels from Stein et al. (2004); OmpF and VDAC from Alvero-González et al. (2024); and Ala-0 and Ala-1 from Queralt-Martín et al. (2018). In **A**, grey solid lines show a scaling *G* ~ *c*^*α*^ with the number indicating the exponent *α*. **D**–**F** Concentration scaling of channel selectivity represented as permeability ratio obtained from reversal potential for several biological and abiotic pores: OmpF and VDAC from Queralt-Martín et al. (2026) and graphene nanopore from Rollings et al. (2016). **A** and **D** panels show neutral channels, **B** and **E** panels display pores with the same outer and inner charges, and **C** and **F** panels feature channels with opposite outer and inner charges. Data have been adapted with permission

Figure 2A illustrates the *G*-*c* relationships in diverse ion channels having in common that all these systems have been reconstituted into neutral membranes (biological ion channels) or are composed of a neutral substrate (synthetic nanopore). A striking observation is the apparent linear relationship between *G* and *c* (*α* ~ 0.5–0.8), which closely mirrors the scaling of solution conductivity (*κ* ~ *c*^0.8–0.9^). This result is even more surprising given that, while the synthetic nanopore is a neutral system, all biological ion channels on display possess ionizable charged residues that confer them noticeable ionic selectivity (especially in diluted solutions). Consequently, one might expect a similar behavior of these charged systems to long synthetic charged nanopores where *G* typically becomes constant in the low-concentration limit, a phenomenon attributed to surface conduction (Stein et al. 2004; Schoch and Renaud 2005; Schoch et al. 2008; Bocquet and Charlaix 2010).

However, many studies show that protein pore charges seem masked because of another contribution that becomes dominant in dilute solutions: the access resistance accounting for the geometrical constriction of the current lines from the bulk solution to a constrained nanochannel (or vice versa) (Aguilella-Arzo et al. 2005; Alcaraz et al. 2017). Crucially, access resistance depends on the solution conductivity near the channel openings, where the inner pore charge regulation is absent. This explains why these intrinsically charged pores may behave as if they were uncharged in terms of conductance scaling (Alcaraz et al. 2017).

One might wonder why access resistance is absent in long synthetic nanopores while this contribution is essential in short biological ones. The key concept here is the nanopore aspect ratio; this is the comparison between the pore diameter *D* and its length *L* (Alcaraz et al. 2017). When *D* <  < *L* (*D* is in the order of nanometers while *L* is micrometric), channel proper resistance is so high that access resistance becomes negligible. However, the advent of new technologies has yielded nanometric pores with lengths comparable to biological bilayers (3–5 nm) or even shorter where access resistance is capital (Lee et al. 2012; Rollings et al. 2016; Aluru et al. 2023; Emmerich et al. 2024).

Interestingly, the access resistance contribution can be tuned such that interfacial effects (*outer* surface effects) either increase or decrease the overall channel conductance. Figure 2B compiles *G*-*c* relationships in charged systems in which *G* almost saturates (*α* ~ 0) in the low-concentration limit attaining values considerably higher than the respective experiment in neutral systems (Queralt-Martín et al. 2018). In all these cases, the membrane charge for the biological channels shares the same sign as the intrinsic net channel charges (e.g., negatively charged membranes for cation-selective channels) (Alcaraz et al. 2017; Queralt-Martín et al. 2018). Similarly, the displayed nanopores feature the same functionalization for the inner and outer pore surfaces. In general, the findings reported in Fig. 2B for both biological and abiotic nanochannels are aligned in showing scaling behaviors constrained to 0 ≤ *α* ≤ 1 (Stein et al. 2004; Amiri et al. 2017; Queralt-Martín et al. 2018; Tao et al. 2021; Noh and Aluru 2023). A recent study in a biological channel, however, demonstrated that a drastic reduction of conductance relative to the neutral case can be achieved in the converse situation (Fig. 2C): when outer (membrane) and inner (pore) charges have opposite signs. In this scenario, the conductance exhibits supralinear scaling (*α* > 1), which is attributed to co-ion-controlled transport (see Equation 14 in  Ref Alvero-González et al. 2024).

The underlying reasons for this diversity of conductance scalings (0 < *α* < 2) lie in the separated charge regulation exerted by both *outer* membrane charges and *inner* pore charges. This has unexpected effects on the concentration scaling of ionic selectivity (Fig. 2D–F). In neutral membranes, the quasi-linear decrease found in conductance with decreasing concentration (Fig. 2A) is accompanied by a quasi-linear increase of selectivity (Fig. 2D). This outcome aligns with classical models where permeability and selectivity operate in a trade-off fashion. A different scenario emerges for charged systems. The increase in conductance observed in Fig. 2B (relative to Fig. 2A) does not come at the cost of reduced selectivity; rather, selectivity actually increases (Fig. 2E). Furthermore, the noticeable decrease in conductance shown in Fig. 2C with respect to Fig. 2A and Fig. 2B is not achieved by increasing selectivity; on the contrary, selectivity decreases (Fig. 2F). This suggests that outer (lipid) charges and inner (protein) charges do not simply compensate for each other but instead function as matching charge clustersof opposite sign (Queralt-Martín et al. 2026).

This peculiar coupling between outer and inner charges can be analyzed using different theoretical approaches, such as equivalent circuits or Poisson-Nernst-Planck models based on the notion of ionic current independence (Alvero-González et al. 2024). Furthermore, by utilizing separate pathways for cations and anions, one can evaluate the ionic selectivity of the system as the ratio of current lines, which again successfully accounts for the experimental findings shown in Fig. 2D–F (Queralt-Martín et al. 2026).

## Concentrated solutions: biphasic concentration patterns

**Fig. 3:**
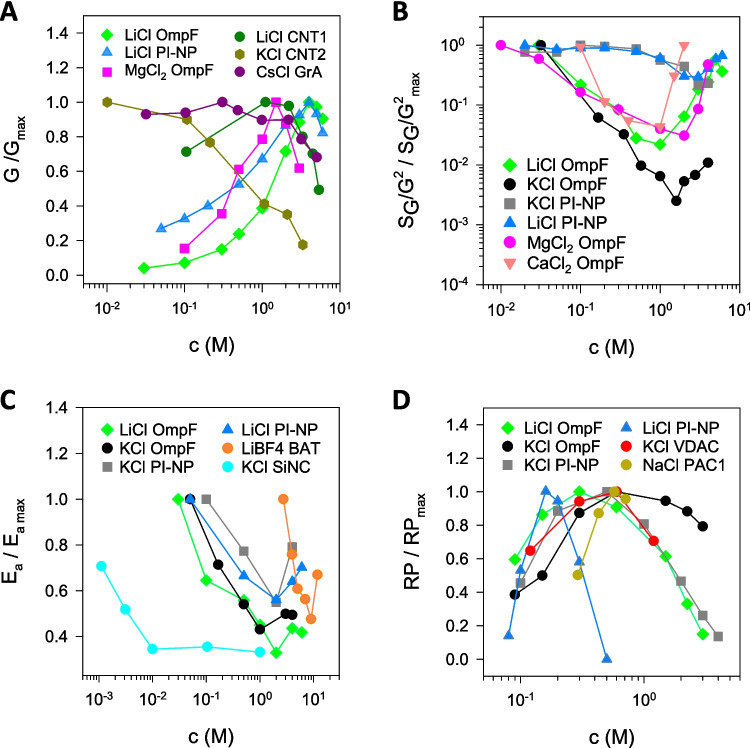
Biphasic concentration scaling under nanoconfinement. Normalized single-channel conductance *G* (**A**), excess noise *S*_G_/*G*^2^ (**B**), activation energy *E*_a_ (**C**), and reversal potential RP (**D**) of diverse systems in the indicated electrolyte. OmpF and PI-NP data from Queralt-Martín et al. (2023); GrA from Rostovtseva et al. (1998); CNT1 from Amiri et al. (2017); CNT2 from Tao et al. (2021); BAT from Doi et al. (2021); SiNC from Ritt et al. (2022); VDAC from Zambrowicz and Colombini (1993); and PAC1 from Cervera et al. (2008). Data have been adapted with permission

Nanoscale ion transport in the high salt concentration regime was addressed in a recent comparative study, which directly put alongside the biological channel OmpF and a synthetic polyimide conical nanopore (PI-NP). It examined the concentration dependence of four key properties: conductance, noise, activation energy, and reversal potential (Queralt-Martín et al. 2023). Here, we gather data from that study together with scattered information from other systems focusing on situations where non-linear effects are observed to illustrate the complexity of concentrated solutions. Figure 3A displays the single-channel conductance of different nanochannels in a variety of electrolytes. Because the actual conductance values span several orders of magnitude, the data are presented as normalized conductances (*G*/*G*_max_). As could be expected given the diversity of pore dimensions, sizes, and conductive properties of these systems, diverse trends emerge. For instance, experiments using LiCl revealed biphasic patterns for both OmpF and PI-NP, with maximal conductance occurring around 5 M (Queralt-Martín et al. 2023), whereas in carbon nanotubes (CNT1) also in LiCl maxima appear at lower concentration (~ 1–2 M) (Amiri et al. 2017). Experiments in gramicidin A (GrA) in CsCl (Rostovtseva et al. 1998) or in OmpF in MgCl_2_ (Queralt-Martín et al. 2015) also show non-linearities with maxima and subsequent decline in highly concentrated solutions. In contrast, experiments with a different type of carbon nanotube (CNT2) in KCl displayed a strictly decreasing pattern (Tao et al. 2021). Importantly, this is the only nanochannel system in which, as far as we know, such behavior appears in conductance measured in KCl (Secchi et al. 2016; Queralt-Martín et al. 2018, 2023; Noh and Aluru 2023). The lack of a universal trend, unlike the established non-linear patterns found in the bulk conductivity of electrolytes (Shcherbakov et al. 2021), suggests that the particular nanoscale confinement, distinctive of each system, is a decisive factor in these conductance measurements. Indeed, diverse mechanisms have been proposed to explain these effects. In carbon nanotubes, the phenomena were attributed to short-time current blockages caused by counterions temporarily adsorbed at the pore surface (Tao et al. 2021) or by the mutual repulsion of ions moving in single-file (Amiri et al. 2017). In gramicidin A in KCl and CsCl, the observations were linked to the existence of multiple binding sites (Rostovtseva et al. 1998; Queralt-Martín et al. 2018). Furthermore, ion-ion repulsion and steric constraints have been cited in the context of narrow, highly occupied potassium channels (Hille and Schwarz 1978).

In addition to channel conductance, complementary information can be obtained while performing the same experiments analyzing the current fluctuations, specifically considering the voltage-dependent excess noise. Most research focuses on the parabolic coefficient *S*_G_/*G*^2^ because it provides crucial kinetic physicochemical information that is not directly observable through *G* measurements (Bezrukov and Winterhalter 2000; Hoogerheide et al. 2009; Queralt-Martín et al. 2015). Observationally, the normalized *S*_G_/*G*^2^ ratio shown in Fig. 3B demonstrates two distinct regimes as a function of salt concentration for the two tested nanochannels (OmpF and NP-PI). The associated inflection points are consistently localized within the *c* ~ 1–2-M range for all electrolytes under study. Previous studies agree in showing that *S*_G_/*G*^2^ experiments in dilute solutions are compatible with surface charge fluctuations (Hoogerheide et al. 2009; Queralt-Martín et al. 2015). However, the rapid raise of normalized *S*_G_/*G*^2^ observed in concentrated solutions has been linked to diverse sources such as binding processes (Queralt-Martín et al. 2015) or transient ion trapping-detrapping at the pore surface (Su et al. 2020).

Importantly, the onset of the high-concentration regime is notably different in the two quantities measured simultaneously in the same experiment, *G* and *S*_G_/*G*^2^. Whereas this transition is undetectable in the conductance measurements done in KCl in both OmpF and NP-PI (Queralt-Martín et al. 2023), it is clearly visible in the corresponding noise experiments (Fig. 3B). Likewise, non-linearities observed in *G* data with LiCl, CaCl_2_ or MgCl_2_ appear at substantially higher concentrations than those observed in the noise data. This disparity leads to the conclusion that the shift between the low- and high-concentration regimes is not universal but is directly influenced by the physical quantity being measured.

In resemblance to *G* and *S*_G_/*G*^2^, similar analyses can be done to the concentration scaling of a key thermodynamic property: the activation energy *E*_a_. Each value of normalized *E*_a_ plotted in Fig. 3C was determined by constructing an Arrhenius plot from conductance measurements across a certain temperature range. While not directly visible in Fig. 3C, the consequences of nanoconfinement are evident: the absolute *E*_a_ values for all presented systems substantially surpass that of the bulk solution conductivity, as shown in previous studies (Alcaraz et al. 2014; Queralt-Martín et al. 2020, 2023). As with the *G* and *S*_G_/*G*^2^ data, these normalized activation energies also display biphasic concentration-dependent trends in OmpF and PI-NP, with inflection points localized around *c* ~ 1–2 M. Comparable biphasic patterns in *E*_a_ showing inflection points in molar concentrations have been reported for Li^+^ transfer across solid electrode-electrolyte interfaces (see Fig. 3C showing results of ref. Doi et al. (2021)), blend polymer electrolytes (Premalatha et al. 2016), and also several different solid polymer electrolytes (Chai and Isa 2013; Aziz et al. 2017; Basha et al. 2018). In these prior studies, the behavior was attributed to distinct mechanisms: association of ions forming clusters (Chai and Isa 2013; Premalatha et al. 2016) and alterations in the solvation structure of concentrated Li^+^ near the electrode interface (Doi et al. 2021). Interestingly, ionic conduction through silica nanochannels showed a kind of saturation at much lower concentration (see Fig. 3C), being ascribed to specific ion adsorption at the pore surface (Ritt et al. 2022).

Non-monotonic trends—as reported in Fig. 3A to C under applied voltage—are also observed in quasi-equilibrium selectivity experiments. The reversal potential (RP), also known as the membrane potential, is defined as the voltage required to produce zero net current across the system when a transmembrane concentration gradient is maintained (Hille 2001) (Laksminarayanaiah and Lakshminarayanaiah 1969) (Helfferich 1962). Figure 3D illustrates normalized RP measurements for various nanochannels as a function of the concentrated side while the diluted one remains constant. Non-monotonic trends are evident in OmpF and PI-NP (Queralt-Martín et al. 2023) as well as in the mitochondrial channel VDAC (Zambrowicz and Colombini 1993) and the meningococcal class 1 porin (Cervera et al. 2008). Similar patterns have been documented in synthetic nanopores (Nishizawa et al. 1995; Guo et al. 2010) and ion exchange membranes (Galama et al. 2016). Previous theoretical studies agree that the initial rise in the absolute RP with an increasing concentration gradient is attributable to electrostatic exclusion. Conversely, the subsequent decrease at higher concentrations is linked to the diffusional contribution that becomes dominant in this regime (López et al. 2009) (Ramirez et al. 2019). This interplay between charge-dominated exclusion (from the channel) and diffusion (linked to intrinsic electrolyte properties) is clearly visible in Fig. 3D. For a given system (OmpF or PI-NP), the position of the maximum RP depends on the specific electrolyte used (KCl or LiCl).

Here we reunite biphasic concentration patterns appearing in electrochemical properties that represent steady-state behavior or quasi-equilibrium states. Remarkably, similar biphasic concentration-dependent patterns also appear in explicitly time-dependent properties like the kinetics of α-cyclodextrin interaction with the ΔCymA channel (Prajapati et al. 2022), the kinetics of voltage-induced gating in the Ompf channel (Alvero-González et al. 2026), or open channel lifetime in channels like the antibiotic peptide gramicidin A (Ring and Sandblom 1988).

## Conclusions

The comprehensive analysis of salt concentration dependence in ion channels reveals two distinct physical regimes that cannot be described with classical models based on Debye screening or ideal-dilute solutions. While such models have proven useful for microscale membrane pores, they fail to capture the unique behavior inherent to nanometer-sized biological and abiotic channels. In dilute solutions, critical interfacial effects reveal a complex relationship between membrane and protein charges, dictating conductance and selectivity scaling with concentration. Such interplay can be explained using the physical concept of ionic current independence, revealing new features overlooked in traditional models. Conversely, data in concentrated solutions shows a departure from Debye screening and the appearance of biphasic patterns in both steady-state and time-dependent quantities due to a complex combination of factors. The inflection point (where the biphasic behavior begins) occurs at a different concentration for every type of measurement (e.g., current, noise, temperature, voltage-induced gating, or selectivity). This non-universal behavior stems from the fact that each experimentally measured property is determined by a unique balance of solvent properties, electrostatic exclusion, ionic diffusion, and ion-ion and ion-pore interactions, all of which are significantly amplified by nanoscale confinement. Remarkably, the concentration at which a given property becomes incompatible with classical Debye screening cannot be reliably predicted by measuring any other property.

These findings have profound implications that extend well beyond membrane biophysics, offering a critical roadmap for the study of biological phenomena in diluted solutions as well as the optimization of next-generation nanotechnology in moderate saline environments. When nanoscale confinement plays a role, standard approximations relying on classical models and the ideality of electrolyte solutions become a limiting factor. Future theoretical frameworks must therefore move beyond these assumptions to bridge the gap between the predictive capabilities of current models and the rich, non-linear reality of ion transport phenomena at the nanoscale.

## Data Availability

No datasets were generated or analysed during the current study.
